# Integrated Palliative Care for Nursing Home Residents: Exploring the Challenges in the Collaboration between Nursing Homes, Home Care and Hospitals

**DOI:** 10.5334/ijic.4186

**Published:** 2019-04-03

**Authors:** Sofie Hermans, Aline Sevenants, Anja Declercq, Nady Van Broeck, Luc Deliens, Joachim Cohen, Chantal Van Audenhove

**Affiliations:** 1 KU Leuven – University of Leuven, LUCAS, Center for Care Research and Consultancy, Minderbroedersstraat, Leuven, BE; 2 KU Leuven – University of Leuven, Department of Clinical Psychology, Tiensestraat, Leuven, BE; 3 End-of-Life Care Research Group, Vrije Universiteit Brussel (VUB) and Ghent University, Laarbeeklaan, Brussels, BE; 4 Department of Internal Medicine, Ghent University, Ghent, BE

**Keywords:** health service integration, integration, palliative care, networks, integrated healthcare systems

## Abstract

**Introduction:**

Nursing home residents are a vulnerable and frail segment of the population, characterised by their complex and palliative care needs. To ensure an integrated approach to palliative care for this target group, working on a collaborative basis with multiple providers across organisational boundaries is necessary. Considering that coordinators of palliative networks support and coordinate collaboration, the research question is: ‘how do network coordinators perceive the process of collaboration between organisations in Flemish palliative networks?’

**Methods:**

A dual-phase sequential mixed-methods design was applied. First, the coordinators of each of the fifteen palliative networks in Flanders completed a survey in which they evaluated ten aspects of collaboration for two types of cooperation: between nursing homes and home care, and between nursing homes and hospitals. Next, the survey results thus obtained were discussed to improve understanding in a focus group composed of the above coordinators, and which was analysed on the basis of content analysis.

**Results:**

In both forms of cooperation, the ‘formalisation’ and ‘governance’ were the aspects that yielded the lowest mean scores. The coordinators in the focus group expressed a need for more formalised interaction among organisations with regard to palliative care, the establishment of formal channels of communication and the exchange of information, as well as the development of shared leadership.

**Conclusions:**

The perspectives of the coordinators on inter-organisational collaboration are a valuable starting point for interventions directed at the stronger integration of palliative care for residents of long term-care facilities.

## Introduction

Patients with complex and long-term care needs are attended to by a wide range of service providers in a variety of care settings. To ensure continuity with regard to the care provided to these patients, the multiple providers involved in this will need to collaborate across organisational boundaries and coordinate their activities [1,2,3,4].

Integration can generally be understood as as bringing together of inputs, delivery, management and organisation of services as a means of improving access, quality, user satisfaction and efficiency [5]. Integration is defined as a coherent set of methods and models on the funding, administrative, organisational, service delivery and the clinical levels designed to create connectivity, alignment and collaboration within and between the cure and care sectors [6]. According to Health Service Research Europe [7], most European countries should improve the integration of multiple services [2,8]. Government policies of Western countries have been increasingly supporting the development of collaborative partnerships in inter-organisational care networks to ensure more integrated care ever since the 1990s. [4,8,9,10,11,12,13,14,15]. In care networks three or more organisations are working toward a common purpose [13]. Either these organisations provide care at the same care level, or organisations providing care at the primary, secondary and tertiary level (such as hospitals, nursing homes and home care organisations) are brought together [5,13].

The importance of inter-organisational collaboration for integrated care is documented in many Western countries in a diversity of areas, such as primary health, mental health, disaster management prevention and early intervention [16,17,18]. In Australia, for example, 31 primary health networks became operational in 2015 in order to integrating public health and primary care in support of the early prevention and coordination of care [19]. In the United States, Canada and Western Europe, policies also aim to integrate care through inter-organisational collaboration [20]. However, the implementation of a care network is no guarantee for successful collaboration, and many organisational collaborations fail in this respect [16,21]. On the basis of their extensive action research on inter-organisational collaboration, Huxham and Vangen [22] conclude that seeking collaborative advantage is a ‘resource-consuming activity’ and inherently difficult. As Auschra puts it, collaboration is a ‘managerial challenge’ [16]. In their literature review, Popp et al. [13] state that there has been excessive focus on network structure in the evaluation of networks, while emphasis should be placed on the processes that lead to the desired outcomes. Successful network functioning relies on the quality and dynamics of the inter-organisational relations that make up the network [18,23]. Much more research is needed on the processes in inter-organisational collaboration that lead to the successful integration of care [7]. The present study aims to analyse the processes of collaboration. This specific case concerns collaboration between organisations providing palliative care for nursing home residents in the palliative networks in Flanders.

## Case Under Analysis

In Flanders (Belgium), palliative networks were implemented in the mid-1990s to support the development of palliative care and improve the coordination of palliative services in a designated region. They are rooted in the informal and voluntary collaboration around palliative home care that emerged in light of a growing awareness of people’s right to receive comprehensive and qualitative end-of-life care. These networks can be seen as umbrella structures under which various organisations offering palliative services (e.g. home care, hospital care, and nursing homes) are brought together with the intention of providing coordinated and good quality palliative care. In 2017, Flanders consist of 15 palliative networks. The accreditation and financial support of these palliative networks is regulated by law [24]. In order to coordinate and control the joint actions of members across the network as a whole, a team with a secretariat, a psychologist and a network coordinator was established. This form of governance structure is labelled a Network Administrative Organisation (NAO) by Provan and Kenis [25]. Among other tasks, it is the responsibility of the NAO to support collaboration among the network members in the region.

Organisational collaboration is particularly important for the integration of palliative care services provided to residents of long term-care facilities. Firstly, during the trajectory in which the patient becomes increasingly dependent on care different care providers and organisations are involved in the provision of palliative care. The admission criteria for nursing home residents focus on the degree of functional limitations, caused by a combination of medical conditions and other patient related and social factors [26,27]. Given that palliative care is not restricted to end-of-life care, important information about the elderly person involved should flow from home care to residential care in order to enhance the comfort of the resident and to safeguard the continuity of care. Furthermore, nursing home residents are often transferred to hospitals and the frequency of these transfers increases toward the end of life [28,29]. Therefore, information should also flow to and from hospitals. To date, the flow of information and communication from one palliative care setting to another is suboptimal [30]. Secondly, research shows the need for palliative care development in nursing homes [31]. Residents of nursing homes tend to be burdened with a vast number of symptoms, are often transferred to hospitals and are not consulted with regard to their end-of-life care preferences [28,29,32,33,34,35].

To optimize the quality of palliative care for nursing home residents, palliative care should become more integrated. Care integration can be examined from the perspective of the processes that underlie inter-organisational collaboration in the network. Given the importance of the integration of palliative care for the nursing home resident, this study aims to analyse the collaborative process among organisations providing palliative care for this target group in the palliative networks in Flanders. The insights obtained by coordinators of palliative networks on the structural and interpersonal aspects of inter-organisational collaboration can help to identify challenges in the integration of palliative care in the Flemish nursing home. The research question in this study is: ‘How do network coordinators perceive the process of collaboration among organisations in Flemish palliative networks?’

## Method

A mixed-methods approach was applied. In a dual-phase study, data was collected sequentially with quantitative and qualitative methods. Phase 1 was a survey study among coordinators of palliative networks and Phase 2 was an additional focus group discussion study with the palliative care coordinators.

### Theoretical framework

The theoretical framework used in this study is the ‘*structuration model of collaboration*’ developed by D’Amour et al. [36]. This model emerged from organisational theory and takes into account both a “meso-structural” level and a “micro” level of collaboration. Both levels (structural – interpersonal) are captured in four dimensions [37,38,39]. Table 1 describes these four dimensions. The framework proved useful in earlier studies to comprehensively analyse collaboration and to identify shortcomings and areas for optimisation of collaboration [18,40,41,42,43,44,45,46].

**Table 1:** Dimensions of the structuration model of collaboration processes.


*Internalisation*	Awareness by professionals of their interdependencies and of the importance of managing them, and which translates into a sense of belonging, knowledge of each other’s values and discipline and mutual trust
*Shared goals and visions*	The existence of common goals and their appropriation by the team, recognition of different motives and multiple allegiances, and the diversity of definitions and expectations regarding collaboration
*Governance*	The leadership functions that support collaboration. Governance gives direction to and supports professionals as they implement innovations related to interprofessional and inter-organisational collaborative practices
*Formalisation*	The extent to which documented procedures that communicate desired outputs and behaviours exist and are being used. Formalization clarifies expectations and responsibilities


Adapted from (A model and typology of collaboration between professionals in healthcare organisations), by D’Amour, D. et al., 2008, *BMC Health Services Research, 8*, 2.

### Setting and participants

The participants of the study are the coordinators of all 15 Flemish palliative care networks. As one network has two coordinators, a total of 16 coordinators were invited to participate. Being experts in palliative care, they are charged with the mission of a palliative network and, in particular, with supporting collaboration between the health care services available in the geographical region covered by the network.

### Data collection

#### Phase 1: Survey research

**Figure 1:**
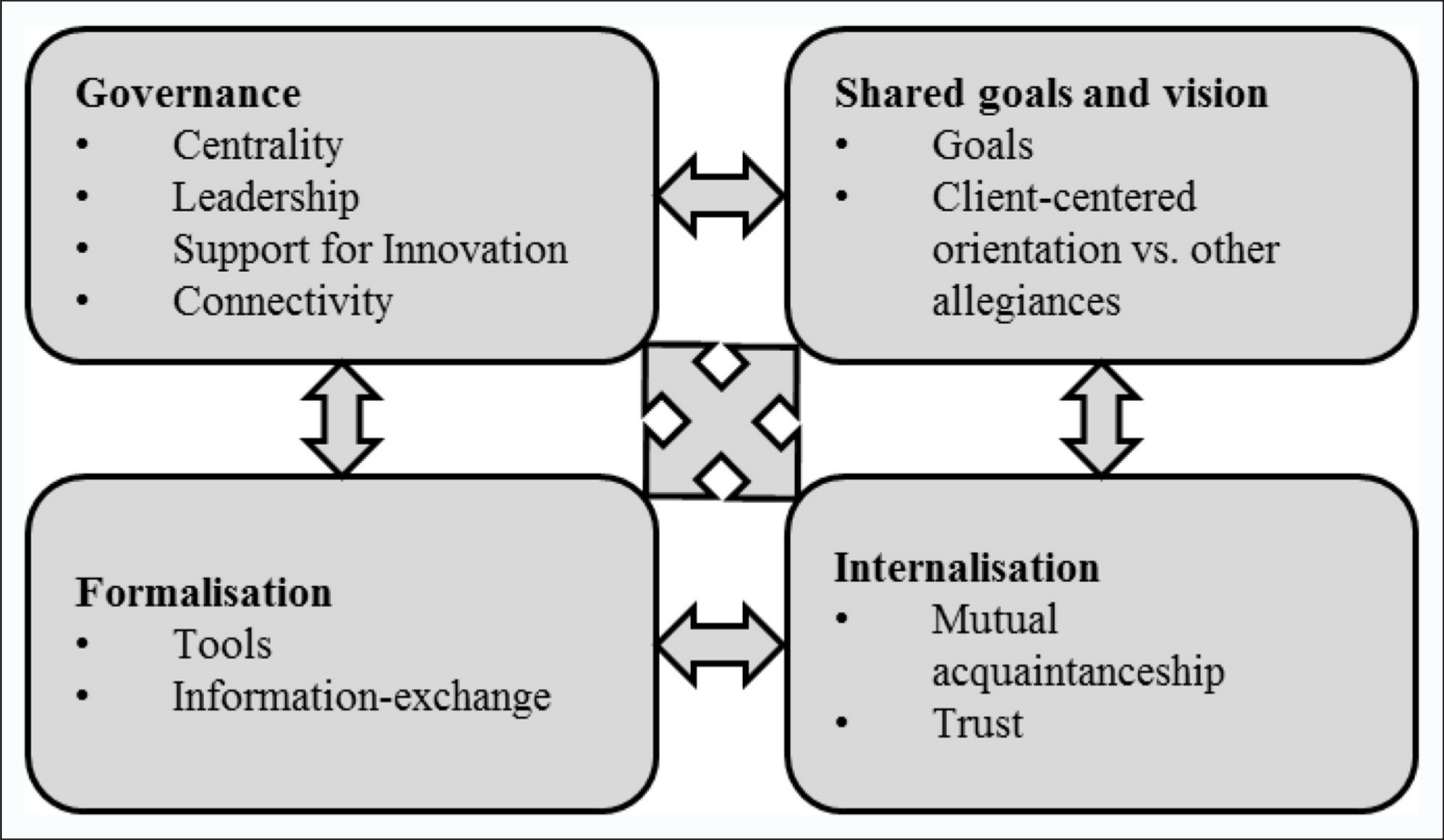
Structuration model of collaboration processes in health care organisations. Adapted from “A model and typology of collaboration between professionals in healthcare organisations”, by D’Amour, D. et al., 2008, *BMC Health Services Research, 8*, 2.

We used a validated questionnaire which is the operationalisation of the “*structuration model of collaboration*”. This survey assesses the degree of collaboration between two types of cooperation in palliative care: nursing homes – home care (1) and nursing homes – hospital care (2). For each type of cooperation, participants were asked to evaluate ten aspects of collaboration in their palliative network on a 5-point Likert scale (see Figure 1). On this scale, 1 corresponds to the lowest degree of collaboration and 5 to the highest. The aspects of collaboration were presented and explained throughout the questionnaire.

The original survey was translated and back-translated from English into Dutch by two independent parties. It was also adapted specifically to the Flemish palliative networks, without changing the propositions on the different aspects of collaboration or the measuring scale, thus preserving the validated characteristics of the questionnaire. Before data collection, the Dutch questionnaire was cognitively tested. The results did not warrant any major changes in the questionnaire. The questionnaires were distributed to the coordinators of the 15 Flemish palliative networks at the end of January 2016. Two weeks later, a reminder was sent. After another two weeks, data had been received from 11 of the 16 palliative network coordinators.

#### Phase 2: Focus group

The focus group composed of coordinators of Flemish palliative networks, started with a presentation of the ‘*structuration model of collaboration*’ and the scores of the survey study in which the coordinators had participated. Next, targeted questions were used to encourage the coordinators to explore those aspects of inter-organisational collaboration in palliative care for nursing home residents that would benefit from optimisation (see Table 2). Co-author C.VA. an experienced moderator and communicator and knowledgeable about the Belgian health care system, acted as discussion leader. She facilitated group discussion by asking for clarification and providing guidance. Also, four members of the research team were present and took notes during the discussion. The focus group discussion was recorded on tape and transcribed.

**Table 2:** Questions during the focus group discussion.


Question 1	Comparing the results of your network with the average score, what are your most important remarks and/or arguments?
Question 2	As stated in the presentation, there is room for optimisation with regard to collaboration between residential care and home care, on the one hand, and collaboration between residential care and hospital care, on the other. According to you, as a coordinator, what is needed to achieve the ideal model of collaboration that D’Amour and colleagues present in Flemish palliative care?


### Data analysis

On the basis of the survey results, the average score on each of the aspects of collaboration was calculated. To gain further insight into how coordinators of palliative networks perceive inter-organisational collaboration in palliative care, a targeted approach to content analysis was used. Data-driven codes created during the initial phase of the analysis were integrated with theory-driven codes in the second phase to enhance trustworthiness [47]. As such, we wanted to diminish the informed bias that stems from using a theory and to overcome blinding by the theory on contextual aspects that influence collaboration among palliative care services (Table 3).

**Table 3:** Representation of data coding.


*Phase 1*	Reading, gaining impressions and gathering thoughts Data-driven coding
*Phase 2*	Theory-driven coding: application of a template of codes by two independent researchers
*Discussion*	Application of the coding template was discussed among two researchers
*Comparison*	Text not categorised in Phase 2 Text categorised in both phases


The analysis was facilitated through the use of QSR NVivo 11 software. In the initial analysis, the text was read, impressions and thoughts were captured in notes and data-driven codes were created. This phase can be considered inductive. In a next step, the 10 predetermined codes of the model were used. The codebook, based on the original definitions of the aspects, was translated into English and subsequently back-translated from English into Dutch by two independent parties (Table 3). In this phase, all text was coded by two independent researchers in the interest of reliability. Subsequently, the coding was discussed by two researchers. Finally, both phases were compared. In this comparison, uncategorised text was reviewed and double-coded text units were investigated to further refine, extend and enrich the results.

### Ethical considerations

The Medical Ethics Committee of Leuven University Hospital was consulted. The Committee concluded that no ethical approval was needed for this study as this study did not require the active involvement of patients or patient information.

## Results

Eleven of the fifteen palliative network coordinators completed the questionnaire, and nine participated in the focus group discussion.

**Figure 2:**
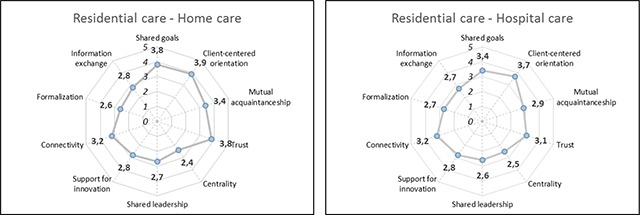
Average scores on aspects of collaboration by coordinators of Flemish palliative networks.

The average scores obtained on various aspects of collaboration provided by the coordinators of Flemish palliative networks are presented as Kiviath graphs in Figure 2. For both partnerships, ‘shared goals’, ‘client-centred orientation’ and ‘trust’ were the aspects to yield the highest average scores. This means that, on average, the coordinators identified some shared goals among organisations in palliative care for nursing home residents. Furthermore, they saw that the preferences and wishes of patients were often taken into account in interaction between settings and that there is a lot of trust among care partners. The lowest scores were obtained with regard to aspects related to the dimensions ‘formalisation’ and ‘governance’. In palliative care for residents of nursing homes, coordinators find few care paths, information systems, protocols or other systems to formalise arrangements regarding the allocation of responsibilities across organisations and little relevant information is exchanged. Furthermore, they indicated that some forums exist in which cross-setting communication takes place, that there is a lack of support for innovation in collaboration and guidelines for collaboration, and that leadership is shared in only a few areas of palliative care for nursing home residents.

### Focus group

The results obtained from the focus group discussion are presented according to the interpersonal and structural dimensions of the model. Most text units were coded in both the data-driven phase and the theory-driven phase of the analysis (see Table 3). Sixteen units of a total of one hundred units are not coded in the second phase, indicating the difficulty of fitting them into the ‘*structuration model of collaboration*’. The content of these text units mainly referred to the organisation of palliative care in Belgium, which relates to the specific context on macro-level in which the model is applied in the present study.

#### Interpersonal dimensions

The palliative network coordinators discussed a lack of personal contact among healthcare workers from different organisations affiliated with the palliative networks. As one coordinator said:

“How often is a homecare nurse contacted for information about a patient upon his or her admission to a nursing home? Hardly ever. How often does a palliative care nurse in a nursing home contact the palliative care team when a patient is transferred to a hospital? In most cases, they just let the patient leave with a letter of referral from the general practitioner. If health practitioners would know each other better, if they can put a face to a name, they would find it easier to contact one another. This would be of great benefit with regard to patient transfer.”

The focus group also agreed that a general consensus about palliative care is difficult to achieve, which is clear from the following statement:

“Even within one and the same nursing home it is difficult to make your own vision on palliative care stick because the various general practitioners who visit their patients in the nursing home let their own vision on palliative care prevail… they do not speak the same language.”

Coordinators talked about the difficulties they experience in motivating key persons to participate in the network’s activities and assigning priority to the general goal of the palliative network – better palliative care.

“In the last evaluation we decided to cancel our joint meetings with the palliative support team and the nursing homes, because the frustration is too big on both sides. We understand each other, we think it’s important and are willing to make time, but the Board of Directors does not want to cooperate. I feel frustrated because I have a low impact on the Board of Directors.”

Several coordinators expressed that personal contact can support other aspects of collaboration, like the exchange of information. Encouraging personal contact and connecting healthcare workers in the palliative field is considered to be a task of the NAO, in which progress is to be made. This is illustrated by the following statement:

“In the last three years the palliative network connected people working in two different health care settings – nursing homes and palliative support teams – through joint workgroups. The aim of putting these groups together was: “If persons of different settings know each other better, information will more easily be shared”. Doing this, I really felt I could fulfil my job as the coordinator of a palliative network.”

Regarding the indicator ‘trust’, coordinators seldom referred to this aspect of collaboration in the focus group and mostly in a negative way:

“What kind of trust is there when we … if information is given when a patient is being transferred and the recipient institution still starts the procedure all over again by asking the patient for the same information again before taking action…”

#### Structural dimensions

Regarding ***formalisation***, the coordinators of palliative networks expressed a need for a formal mechanism with regard to the exchange of information when a nursing home resident is transferred from one setting to another. The coordinators were in agreement that the exchange of information in such cases should be representative of all health professionals involved in the palliative care of nursing home residents and the patients themselves. In that way, a comprehensive perspective on the patients’ condition can be attained.

“When a patient is transferred from home care to residential care, there is never enough consultation between the various settings. A family can add information about the elderly family member to a list, but in this admission process, no other health professionals are involved.”

They reported that patients have to give delicate information regarding their situation and preferences again when they are transferred to a new setting. They talked about advance care planning as a way to formally incorporate the treatment preferences of the patient in the transfer process. As one of the coordinators stated:

“Actually, admission in a nursing home should be a consequence of advanced care planning. In this plan, the person can stipulate that it is his or her preference to be admitted when living at home is no longer a sustainable option.”

They discussed that they would like to see inter-organisational agreements being made that facilitate advance care planning.

“I think something very concrete for us is the complaint you often hear in nursing homes regarding advance care planning. Actually, advance care planning should accompany the patient from the home care setting. For me, this topic could be part of ‘shared leadership’, meaning that there would be formal arrangements between home care and nursing homes regarding the transfer of advance care planning from home care to residential care.”

In the ***governance*** of inter-organisational collaboration, the coordinators stated that they miss clear guidelines for collaborative action. They also talked about a lack of innovation with regard to a collaboration-based approach to palliative care and shared leadership. Furthermore, they found that connectivity could be improved.

According to the coordinators innovative changes in the culture and attitudes regarding palliative care are made possible when organisational leaders direct the actions of healthcare workers in their own organisation. They stated that innovative changes like these are hampered by financial constraints at organisational level. To provide clear guidance, the coordinators think that the government could take measures to stimulate organisations towards embracing collaboration (e.g. by rewarding inter-organisational communication with regard to the transfer of patients by giving them additional funding, or providing for control measures to make collaboration compulsory). In the next statement, a coordinator clearly states that financial incentives can regulate collaboration in palliative care:

“Perhaps the government of Flanders should provide financial compensation for the setting up of meetings with care partners in the region in its current financial model. I think Flanders will implement such funding in the future.”

Furthermore, the coordinators discussed that two aspects of the prevailing legal design and operation of the palliative networks hampers collaborative behaviour of organisations. First, collaboration between settings in the palliative network is not mandatory and second, although some financial reward is granted by the government, all organisations are free to enter into a cooperation commitment with the network.

“The caring people in the managing boards of nursing homes and hospitals who initially established the organisations are increasingly replaced by hardcore business managers who think primarily in figures. As a consequence, it is more difficult for health staff to participate in training or working groups because it is too expensive and too time-consuming.”

Also, the coordinators often mention doctors as ‘key figures’ in the collaboration of palliative care services because Belgian law gives them a lot of responsibility. They associate the medical profession with power and struggle to persuade them of the importance of palliative care.

“I would even consider this a level below that of the Board of Directors. In distinguishing between nursing homes, the management, medical staff and hospitals… it is proven that hospital doctors are the least palliative-minded of all members of the medical profession. In a nursing home, convincing the coordinating general practitioner of a palliative approach will have a positive effect on the Board of Directors. But in a hospital, many doctors are very difficult to persuade and the Medical Board has a strong influence on hospital policy.”

The coordinators talked about shared leadership as the coming together of persons of different organisations who have “…a mandate to modify or debate certain issues…” that play a role in inter-organisational collaboration and to reach agreements and develop procedures. Although differences between networks were noted in the focus group with regard to the frequency with which the member organisations meet and speak about the vision and mission of palliative care, the coordinators generally experience difficulties in gathering leaders around the table. They referred to an imbalance in power between the organisations:

“We have to fight to stand next to the director and to discuss such matters. On a clinical level, bed-side, this is not a problem. On this ‘individual level’, we are more than welcome to approach the persons who take care of the patient. But on a policy level it is difficult to broach the subject of palliative care. I often feel that the palliative network is not taken seriously as an organisation. You really have to fight and put yourself forward politically to get things done.”

More specifically, the palliative network coordinators reported encountering problems with organising meetings, motivating the key persons of the various organisations to attend meetings and focus sufficiently on palliative care, and confronting key persons with problems regarding palliative care. Nevertheless, they spoke about the importance of involving professionals from different healthcare facilities for the provision of qualitative palliative care. The coordinators agreed that ‘bringing people together’ in joint meetings, working committees or consultative forums is a task of the network’s NAO. The following statement illustrates this:

“As a network we have the opportunity or the potential to connect people, an aspect of collaboration in which there is still a lot of progress to be made. I think we can make the transfer of palliative patients more efficient if we initiate regular contact between persons who usually work together: palliative care professionals from nursing homes and people from the palliative support team in hospitals. We should promote the exchange of information and clear contact. I think we could benefit tremendously from this.”

## Discussion

This study leads to greater insight into how coordinators of palliative networks perceive collaboration among healthcare organisations involved in providing palliative care to nursing home residents. In line with the results of the survey that preceded the focus group, coordinators of palliative networks stress that there is room for improvement with regard to the dimensions linked to the structural aspects of collaboration: governance and formalisation. Although these results refer to a unique challenge for the Belgian health care system, they also seem to be consistent with some general findings in the literature about health care integration.

In the literature, *formalisation* is identified as an essential aspect in effective collaboration [48,49,50,51]. The present study highlights the lack of the organised, systematic exchange of information in which all parties (patients and caregivers from different settings, such as general practitioners, home care nurses, specialists, etc.) involved in the care process, are included. To the coordinators of palliative networks, advance care planning is a promising way to take the treatment preferences of nursing home residents into account through this inter-sectoral exchange of information. Studies have indeed shown that residents of long-term care facilities benefit from advance care planning: their wishes and preferences are better known and respected [52,53,54]. However, the implementation of advance care planning in nursing homes can be further improved [53]. More studies are required to investigate the effect of an advance care plan on the goals of care provided in the organisations were the patient is transferred to (like an emergency ward) [55].

With respect to the structural dimension ‘*governance*’, coordinators of palliative networks report difficulties with regard to ‘*shared leadership*’, ‘*support for innovation*’ and ‘*centrality*’. Research shows the importance of this dimension, and the importance of ‘*leadership*’ in particular, in the effective functioning of collaborative undertakings [13,45,48,56]. Coordinators of palliative networks state that *shared leadership* or joint decision-making is a responsibility of persons who have ‘*a mandate to modify*’. Two case studies in healthcare show that implementing change involving integration across organisational boundaries indeed requires the dispersion of authority, resources and expertise [57,58]. Therefore, shared leadership is a crucial factor if changes are to be made in favour of integrating palliative care for older persons. Other studies on palliative care networks yield the same conclusion and indicate a clear need for leadership development [51,59].

The structure of a collaborative undertaking is often externally imposed by policy makers and determines which participants can take up a leadership position [60,61]. The palliative networks established by the Belgian government are designed in such a way that coordinators and the Board of Directors are expected to play a leadership role. The results of the present study show that coordinators of palliative networks consider themselves responsible for bringing people together under the banner of palliative care. However, difficulties in gaining the attention of key persons and motivating them have left coordinators of palliative networks with a sense of powerlessness. This could explain why coordinators of palliative networks miss adequate government support in ‘*getting the right people around the table*’ and in concluding inter-organisational agreements to formalise the commitment of the various organisations involved in palliative care. The lack of interest in and dedication to palliative care exhibited by key persons might be linked to high staff turnover among this population. A high turnover is associated with the lack of specialised knowledge about palliative care and a lack of support for palliative care within the ranks of the organisation [62,63].

In the literature, difficulties in leading or managing collaborative structures are acknowledged [13,25,61,64]. In line with our results, especially mobilising important key persons is proven to be a challenging aspect of their role [65]. Popp and colleagues [13] state that leaders within a networked environment should ‘*nurture shared leadership or decision-making*’, denoted with the terms ‘facilitator’, ‘broker’ or ‘boundary spanner’. Interpersonal skills are primary competencies for network leaders, which is not surprising given the fact that relationships between actors (organisations and individuals) form the foundation of collaborative partnerships [13,64,66]. The responsibility of leaders is to establish a common ground and build credibility and trust [67]. Consequently, leaders should act on identifying a common rationale for collaboration (shared goals and vision) and create awareness of mutual interdependencies (internalisation), which are dimensions of the micro level of collaboration.

Results of the survey indicate that coordinators perceive a reasonable amount of trust between the members of the various organisations. However the coordinators of palliative networks in the focus group do not often refer to the indicator ‘trust’. This finding is contradictory with the existing literature, proving that trust plays a critical role in the success of a collaboration project [13]. Popp et al. [13] describe trust (p. 19) as “the lubricant” of a network, indicating its necessity. Network management plays an important role in building relationships of trust through the growth of a network. Moreover, in this review trust is linked to knowing each other (mutual knowledge), the willingness to share information (the exchange of information) and achieving goal consensus (shared goals). Although it is a strength of the model of D’Amour et al. [36] that both levels of collaboration are incorporated in our understanding of the process of collaboration, the interrelation between aspects is not specified. Further research and possible interventions could account for links between aspects of collaboration in palliative care.

### Strengths and limitations

One of the strengths of the present study is the use of the validated “structuration model of collaboration”. This has the advantage of providing an approach to tackle the complex issue of collaboration between networks in different countries and societal contexts, and to elicit strengths and challenges in this collaboration that can further guide possible intervention.

Furthermore, this case study contributes to the development and improvement of measures for collaboration because there are only a limited number of adequate instruments available to evaluate inter-organisational collaboration. The adapted instrument of this case study can be used to examine inter-organisational collaboration in healthcare contexts other than palliative care and contexts other than the Belgian-Flemish community. In a recent study by Meijer et al. [68] in the Netherlands the instrument developed by Nuño-Solínis et al. [42] was also translated and adapted. These authors found the instrument to be inadequate in terms of validity and reliability and warn against its use in a different cultural setting, population, or context without testing it first. We agree that merely using the instrument in quantitative research is not flawless and should not be recommended. However, a triangulation of qualitative and quantitative data does offer better insight into problem areas in inter-organisational collaboration. We believe that this mixed-methods approach represents an important initial step in the examination of inter-organisational collaboration in care networks. Certain limitations arise in the trustworthiness of this mixed-methods research [47,69,70]. The use of the “structuration model of collaboration” could have biased the data analysis in that other aspects for inter-organisational collaboration are not taken into account. However, several measures were taken to benefit the credibility and confirmability of this research. Firstly, the use of an iterative analysis process allowed for non-supporting evidence to emerge out of the qualitative data. Codes that didn’t ‘fit’ the model, were thoroughly discussed with two other researchers. Secondly, data triangulation was optimized through the integration of quantitative and qualitative methods of investigation. Although it was not intended to use the data from the survey investigation for quantitative analysis, the data formed a point of reflection throughout the research process regarding collaboration. Also, a satisfying amount of coordinators of palliative networks participated in the focus group. Proportionally, they covered a large amount of palliative networks and provided information-rich data. Furthermore, a second researcher who did not take part in the focus group coded the data and gave input in the discussion on the coding, thus providing for an external check on the research.

### Conclusion

In several countries and health domains, governments aim to integrate the provision of care by creating care networks. Based on this case study, the following policy recommendations can be made. First of all, the implementation of a care network does not guarantee successful collaboration between care organisations. In this mixed-method study, the coordinators of palliative care networks identify the need to formalise the interaction between various health care organisations with regard to palliative care, the establishment of formal channels of communication and information exchange, as well as the development of shared leadership. Secondly, when designing care networks, leadership positions should be taken into consideration. Given the difficulties network coordinators encounter with shared leadership, interpersonal skills training could be of benefit to their professional development. Also, incentives could be put forward to motivate key persons to attend meetings. As mentioned in the review by Muller-Seitz [71], research needs to contribute more to the current understanding of how leadership is ‘made to happen’. Future studies could validate the results by exploring the views of ‘key persons’ on inter-organisational collaboration in networks, leading to a more comprehensive understanding of the challenges in collaboration.

## References

[1] Anderson, GF and Hopkins, J. For 50 years OECD countries have continually adapted to changing burdens of disease; the latest challenge is people with multiple chronic conditions Paris: OECD Publishing; 2011 Une. [cited 2017 8 Nov]. Available from: http://www.oecd.org/els/health-systems/48130653.pdf.

[2] Kodner, DL. All together now: A conceptual exploration of integrated care. Healthc Q, 2009; 13: 6–15. DOI: 10.12927/hcq.2009.21091 20057243

[3] Botezat, D, Oprea, L and Gavrilovici, C. The integration of Health Services. Health Networks. Social Research Reports, 2013; 23: 68–81. www.reasearchreports.ro.

[4] Schoen, C, Osborn, R, How, SKH, Doty, MM and Peugh, J. In Chronic Condition: Experiences Of Patients With Complex Health Care Needs, In Eight Countries. Health Affairs, 2009; 28(1): 1–16. DOI: 10.1377/hlthaff.28.1.w1 19008253

[5] Grone, O and Garcia-Barbero, M. Integrated care: A position paper of the WHO European Office for Integrated Health Care Services. Int J Integr Care, 2001; 1: 1–21. http://www.ijic.org/ DOI: 10.5334/ijic.28 PMC1525335 16896400

[6] Kodner, D and Spreeuwenberg, C. Integrated care: Meaning, logic, applications, and implications – a discussion paper. Int J Integr Care, 2002; 2 http://www.ijic.org/ DOI: 10.5334/ijic.67 PMC1480401 16896389

[7] HSR-Europe. Health services research: Helping tackle Europe’s health care challenges (policy brief) Utrecht: NIVEL; 2010 12 [cited 2018 19 Jan]. Available from: https://www.nivel.nl/sites/default/files/bestanden/HSR-Europe--2010--Health_services_research--Helping_tackle_Europe%27s_health_care_challenges--Draft_Policy_Brief.pdf.

[8] Goodwin, N, Smith, J, Davies, A, Perry, C, Rosen, R, Dixon, A, et al. Integrated care for patients and populations: Improving outcomes by working together London: The Kings’s Fund; 2012 [cited 2017 23 Nov]. Available from: https://www.kingsfund.org.uk/.

[9] World Health Organization. World Health Report: Primary Health Care (Now More Than Ever); 2008 Available from: http://www.who.int/whr/2008/chapter1/en/.

[10] Agentschap Zorg en Gezondheid. Een geïntegreerde zorgverlening in de eerste lijn in Conferentie eerstelijnszorg [Conferention of Integrated Care in the Community], Agentschap Zorg en Gezondheid (eds.). Agentschap Zorg & Gezondheid: EGG Brussel; 2017 https://www.zorg-en-gezondheid.be/ [in Dutch].

[11] Evans, JM, Baker, GR, Berta, W and Barnsley, J. The evolution of integrated health care strategies. Advances in Health Care Management, 2013; 15: 125–161. DOI: 10.1108/S1474-8231(2013)0000015011 24749215

[12] Cohen, J, Smets, T, Pardon, K and Deliens, L. Palliatieve zorg, meer dan stervensbegeleiding [Palliative Care: More than End-of-Life Care]. Leuven: LannooCampus; 2014. [in Dutch].

[13] Popp, JK, Milward, BH, McKean, G, Casebeer, A and Lindstrom, R. Inter-Organizational Networks: A Review of the Literature to Inform Practice Edmonton, Alberta: Alberta Centre for Child, Family and Community Research Available from: http://www.businessofgovernment.org/.

[14] Cunningham, FC, Ranmuthugala, G, Westbrook, JI and Braithwaite, J. Net benefits: Assessing the effectiveness of clinical networks in Australia through qualitative methods. Implementation Science, 2012; 7 DOI: 10.1186/1748-5908-7-108 23122000 PMC3541150

[15] Van den Heuvel, B. Netwerkzorg: een nieuw organisatieconcept voor personen met een complexe, langdurige zorg- en ondersteuningsvraag [Care Networks: A new organisational concept for persons with complex and chronic care needs] Leuven: ACCO; 2014. [in Dutch].

[16] Auschra, C. Barriers to the Integration of Care in Inter-Organisational Settings: A Literature Review. International Journal of Integrated Care, 2018; 18: 14 DOI: 10.5334/ijic.3068 PMC5887071 29632455

[17] Valaitis, R, McCarthy, J, Macdonald, M, Wong, S, Martin-Misener, R and Akhtar-Danesh, N. Strengthening primary health care through primary care and public health collaboration Ottowa: CFHI; 2012 12 [cited 2017 10 Dec]. Available from: http://www.cfhi-fcass.ca/Libraries/Reports/Strengthening-Primary-HealthCare-Dec2012-E.sflb.ashx.

[18] D’Amour, D, Goulet, L, Pineault R, Labadie J-F and Remondin, M. Comparative study of interorganizational collaboration in four health regions and its effects: The case of perinatal services Montréal, Canada; 2003 6 [cited 2018 20 Jan]. Available from: http://www.ferasi.umontreal.ca/fra/07_info/Rapport%20ANG.pdf.

[19] Booth, M, Hill, G, Moore, JM, Dalla, D, Moore, GM and Messenger, A. The new Australian Primary Health Networks: How will they integrate public health and primary care? Public Health Research & Practice, 2016; 26(1): 1–5. DOI: 10.17061/phrp2611603 26863166

[20] Mossialos, E, Djordjevic, A, Osborn, R and Sarnak, D. International Profiles of Health Care Systems. New York: The Commonwealth Fund; 2017 5 [cited 2018 20 Jan]. Available from: http://international.commonwealthfund.org/features/integration/.

[21] Bainbridge, D, Brazil, K, Krueger, P, Ploeg, J, Tanigichi, A and Darnay, J. Measuring healthcare integration: Operationalization of a framework for a systems evaluation of palliative care structures, processes, and outcomes. Palliative Medicine, 2016; 30(6): 567–579. DOI: 10.1177/0269216315619862 26934948

[22] Huxham, C and Vangen, S. Managing to collaborate: The theory and practice of collaborative advantage. London: London Routledge; 2005.

[23] Alison, G. Maintaining Relationships Is Critical in Network’s Success. Health care Papers, 2006; 7(2): 28–31. 17167315

[24] Federatie Palliatieve Zorg Vlaanderen. Wat is een netwerk palliatieve zorg? [What is a Palliative Care Network?] [webpage on the internet]. [cited 2017 4 Nov updated 2017 May 9]. Available from: http://www.palliatief.be/netwerken [in Dutch]. DOI: 10.12927/hcpap..18553

[25] Provan, KG and Kenis, P. Modes of network governance: Structure, management, and effectiveness. Journal of Public Administration Research and Theory, 2008; 18(2): 229–252. DOI: 10.1093/jopart/mum015

[26] Van Rensbergen, G and Nawrot, T. Medical Conditions of Nursing Home Admissions. Bmc Geriatrics, 2010; 10: 9 DOI: 10.1186/1471-2318-10-46 20630079 PMC2912913

[27] Van Rensbergen, G and Pacolet, J. Instrumental Activities of Daily Living (I-ADL) trigger an urgent request for nursing home admission. Archives of Public Health, 2012; 70: 8 DOI: 10.1186/0778-7367-70-2 22958483 PMC3415109

[28] Van Rensbergen, G, Nawrot, T, Van Hecke, E and Nemery, B. Where do the elderly die? The impact of nursing home utilisation on the place of death. Observations from a mortality cohort study in Flanders. BMC Public Health, 2006; 6: 178–178. DOI: 10.1186/1471-2458-6-178 16824222 PMC1552067

[29] Block van den, L, Pivodic, L, Pardon, K, Donker, G, Miccinesi, G, Moreels, S, et al. Transitions between health care settings in the final three months of life in four EU countries. European Journal of Public Health, 2015; 25(4): 569–575. DOI: 10.1093/eurpub/ckv039 25829502

[30] Firn, J, Preston, N and Walshe, C. What are the views of hospital-based generalist palliative care professionals on what facilitates or hinders collaboration with in-patient specialist palliative care teams? A systematically constructed narrative synthesis. Palliative Medicine, 2016; 30(3): 240–256. DOI: 10.1177/0269216315615483 26873984

[31] Froggatt, K, Payne, S, Morbey, H, Edwards, M, Finne-Soveri, H and Gambassi, G. Palliative Care Development in European Care Homes and Nursing Homes: Application of a Typology of Implementation. J Am Med Dir Assoc, 2017; 18(6): 550.e7–550.e14. DOI: 10.1016/j.jamda.2017.02.016 PMC5754324 28412166

[32] Smedback, J, Ohlen, J, Arestedt, K, Alvariza, A, Furst, CJ and Hakanson, C. Palliative care during the final week of life of older people in nursing homes: A register-based study. Palliative & Supportive Care, 2017; 15(4): 417–424. DOI: 10.1017/S1478951516000948 28049547

[33] Estabrooks, CA, Hoben, M, Poss, JW, Chamberlain, SA, Thompson, GN, Silvius, JL and Norton, PG. Dying in a Nursing Home: Treatable Symptom Burden and its Link to Modifiable Features of Work Context. Journal of the American Medical Directors Association, 2015; 16(6): 515–520. DOI: 10.1016/j.jamda.2015.02.007 25805625

[34] Thompson, GN, Doupe, M, Reid, RC, Baumbusch, J and Estabrooks, CA. Pain Trajectories of Nursing Home Residents Nearing Death. Journal of the American Medical Directors Association, 2017; 18(8): 700–706. DOI: 10.1016/j.jamda.2017.03.002 28431914

[35] De Roo, ML, Albers, G, Deliens, L, de Vet, HCW, Francke, AL, Van den Noortgate, N, et al. Physical and Psychological Distress Are Related to Dying Peacefully in Residents With Dementia in Long-Term Care Facilities. Journal of Pain and Symptom Management, 2015; 50(1): 1–8. DOI: 10.1016/j.jpainsymman.2015.02.024 25847852

[36] D’Amour, D, Goulet, L, Labadie, JF, Martin-Rodriguez, LS and Pineault, R. A model and typology of collaboration between professionals in healthcare organizations. BMC Health Services Research, 2008; 8: 14 DOI: 10.1186/1472-6963-8-188 18803881 PMC2563002

[37] Crozier, M and Friedberg, E. L’acteur et le système. Les contraintes de l’action collective [The actor and the system: Barriers of collective action]. Paris: Seuil; 1977. [in French].

[38] Friedberg, E. Le Pouvoir et la Règle: Dynamiques de l’action organisée [The Multimedia Encyclopedia of Organization Theory]. Paris: Seuil; 1993. [in French].

[39] D’Amour, D, Sicotte, C and Levy, R. Collective Action within Interprofessional Teams in Health Services. Sciences Sociales et Santé, 1999; 17(3): 1–14.

[40] Daigle, K. La collaboration entre les infirmières d’un centre hospitalier (CH) et d’un centre local de services communautaires (CLSC) dans le cadre du congé précoce en postnatal [Collaboration between hospital nurses and nurses from a community centre regarding early discharge and prenatal care]. Montréal: Université de Montréal; 2000. [in French].

[41] Camara, MD, Paino, M, Vinas, I, Arteagoitia, M, Zabala, I and Sola, C. Evaluating Integrated Care in the Basque Country: Using IEMAC-ARCHO and D’Amour Questionnaire. International Journal of Integrated Care, 2016; 16: 3 DOI: 10.5334/ijic.2825

[42] Nuno-Solinis, R, Berraondo Zabalegui, I, Sauto Arce R, San Martin Rodriguez, L and Toro Polanco, NR. Development of a questionnaire to assess interprofessional collaboration between two different care levels. International journal of integrated care, 2013; 13: 1–12. http://www.ijic.org DOI: 10.5334/ijic.984 PMC3718263 23882165

[43] Nuno-Solinis, R, Zabalegui, IB, Rodriguez, LS, Arce, RS and Gagnon, MP. Does interprofessional collaboration between care levels improve following the creation of an integrated delivery organisation? The Bidasoa case in the Basque Country. Int J Integr Care, 2013; 13: 1–11. http://www.ijic.org DOI: 10.5334/ijic.1118 PMC3812313 24179454

[44] Polanco, NT, Zabalegui, IB, Irazusta, IP, Solinis, RN and Camara, MD. Building integrated care systems: A case study of Bidasoa Integrated Health Organisation. International Journal of Integrated Care, 2015; 15: 13 http://www.ijic.org DOI: 10.5334/ijic.1796 PMC4491322 26150764

[45] San Martin-Rodriguez, L, Beaulieu, MD, D’Amour, D and Ferrada-Videla, M. The determinants of successful collaboration: A review of theoretical and empirical studies. J Interprof Care, 2005; 19(1): 132–47. DOI: 10.1080/13561820500082677 16096151

[46] D’Amour, D, Goulet, L, Pineault, K and Daigle, K. Strategic analysis of the implementation of a network of perinatal care In: Pineault, R and Tousignant, P (Eds.), Transformation of the Montreal network: Impact of health, 2001; 53–59. Montréal, Canada: RRSSSM-C.

[47] Hsieh, HF and Shannon, SE. Three approaches to qualitative content analysis. Qualitative Health Research, 2005; 15(9): 1277–1288. DOI: 10.1177/1049732305276687 16204405

[48] McInnes, E, Haines, M, Dominello, A, Kalucy, D, Jammali-Blasi, A and Middleton, S. What are the reasons for clinical network success? A qualitative study. BMC Health Services Research, 2015; 15(1): 2–9. DOI: 10.1186/s12913-015-1096-5 PMC4635586 26541410

[49] Nylen, U. Interagency collaboration in human services: Impact of formalization and intensity on effectiveness. Public Administration, 2007; 85(1): 143–166. DOI: 10.1111/j.1467-9299.2007.00638.x

[50] Willem, A and Gemmel, P. Do governance choices matter in health care networks? an exploratory configuration study of health care networks. BMC Health Services Research, 2013; 13: 1–10. DOI: 10.1186/1472-6963-13-229 23800334 PMC3727985

[51] Albers, G, Froggatt, K, Van den Block, L, Gambassi, G, Vanden Berghe, P, Pautex, S, et al. A qualitative exploration of the collaborative working between palliative care and geriatric medicine: Barriers and facilitators from a European perspective. BMC Palliative Care, 2016; 15: 1–10. DOI: 10.1186/s12904-016-0118-3 27169558 PMC4866297

[52] Kirsebom, M. Mind the gap. Organizational factors related to transfers of older people between nursing homes and hospital care Uppsala: Faculty of Medicine; 2015 [cited 2017 5 Dec]. Available from: http://www.diva-portal.org/smash/record.jsf?pid=diva2%3A843829&dswid=-9872.

[53] Gilissen, J, Pivodic, L, Smets, T, Gastmans, C, Vander Stichele, R, Deliens, L, et al. Preconditions for successful advance care planning in nursing homes: A systematic review. International Journal of Nursing Studies, 2017; 66: 47–59. DOI: 10.1016/j.ijnurstu.2016.12.003 27987411

[54] Martin, RS, Hayes, B, Gregorevic, K and Lim, WK. The Effects of Advance Care Planning Interventions on Nursing Home Residents: A Systematic Review. Journal of the American Medical Directors Association, 2016; 17(4): 284–293. DOI: 10.1016/j.jamda.2015.12.017 26861748

[55] Street, M, Ottmann, G, Johnstone, MJ, Considine, J and Livingston, PM. Advance care planning for older people in Australia presenting to the emergency department from the community or residential aged care facilities. Health & Social Care in the Community, 2015; 23(5): 513–522. DOI: 10.1111/hsc.12162 25443161

[56] Short, A, Phillips, R, Nugus, P, Dugdale, P and Greenfield, D. Developing an inter-organizational community-based health network: An Australian investigation. Health Promotion International, 2015; 30(4): 868–880. DOI: 10.1093/heapro/dau021 24760546

[57] Chreim, S, Williams, BE, Janz, L and Dastmalchian, A. Change agency in a primary health care context: The case of distributed leadership. Health Care Management Review, 2010; 35(2): 187–199. DOI: 10.1097/HMR.0b013e3181c8b1f8 20234224

[58] Buchanan, DA, Addicott, R, Fitzgerald, L, Ferlie, E and Baeza, JI. Nobody in charge: Distributed change agency in healthcare. Human Relations, 2007; 60(7): 1065–1090. DOI: 10.1177/0018726707081158

[59] Hasselaar, J and Payne, S. Integrated Palliative Care – InSup-C1. Nijmegen: Radboud University Center; 2016.

[60] Crosby, BC and Bryson, JM. A leadership framework for cross-sector collaboration. Public Management Review, 2005; 7(2): 177–201. DOI: 10.1080/14719030500090519

[61] Huxham, C and Vangen, S. Leadership in the shaping and implementation of collaboration agendas: How things happen in a (not quite) joined-up world. Academy of Management Journal, 2000; 43(6): 1159–1175. DOI: 10.2307/1556343

[62] Tilden, VP, Thompson, SA, Gajewski, BJ and Bott, MJ. End-of-Life Care in Nursing Homes: The High Cost of Staff Turnover. Nursing Economics, 2012; 30(3): 163–166. 22849015

[63] van Riet Paap, J, Vernooij-Dassen, M, Brouwer, F, Meiland, F, Iliffe, S, Davies, N, Leppert, W, Jaspers, B, Mariani, E, Sommerbakk, R, Vissers, K and Engels, Y. Improving the organization of palliative care: Identification of barriers and facilitators in five European countries. Implementation science: IS, 2014; 9: 130 DOI: 10.1186/s13012-014-0130-z 25686479 PMC4203898

[64] Jang, HS, Valero, JN and Jung, K. Effective Leadership in Collaboration: Lessons Learned from Continuum of Care Homeless Programs. Washington DC: IMB Center for The Business of Government; 2016 [cited 2017 17 Dec]. Available from: http://www.businessofgovernment.org.

[65] Kousgaard, MB, Joensen, ASK and Thorsen, T. The challenges of boundary spanners in supporting inter-organizational collaboration in primary care – a qualitative study of general practitioners in a new role. Bmc Family Practice, 2015; 16: 1–9. DOI: 10.1186/s12875-015-0231-z 25887910 PMC4355472

[66] Silvia, C and McGuire, M. Leading public sector networks: An empirical examination of integrative leadership behaviors. Leadership Quarterly, 2010; 21(2): 264–277. DOI: 10.1016/j.leaqua.2010.01.006

[67] Williams, P. Special Agents: The Nature and Role of Boundary Spanners In: Collaborative Futures: New Insights from Intra and Inter-Sectoral Collaborations. Birmingham: University of Birmingham; 2010 Febr [cited 2018 15 Jan]. Available from: http://www.researchcatalogue.esrc.ac.uk/grants/RES-451-26-0672/read.

[68] Meijer, L, de Groot, E, van Smeden, M, Schellevis, FG and Damoiseaux, RAMJ. Challenges in measuring interprofessional–interorganisational collaboration with a questionnaire. BJGP Open, 2018: 1–9. DOI: 10.3399/bjgpopen18X101385 PMC6181086 30564705

[69] Netwerk Kwalitatief Onderzoek AMC – UvA. Richtlijnen voor kwaliteitsborging in gezondheids(zorg)onderzoek: Kwalitatief Onderzoek [Guidelines for quality in healthcare research: Qualitative Research]. Amsterdam: AMC-UvA; 2002. [in Dutch].

[70] Malterud, K. Qualitative research: Standards, challenges, and guidelines. Lancet, 2001; 358(9280): 483–488. DOI: 10.1016/S0140-6736(01)05627-6 11513933

[71] Muller-Seitz, G. Leadership in Interorganizational Networks: A Literature Review and Suggestions for Future Research. International Journal of Management Reviews, 2012; 14(4): 428–443. DOI: 10.1111/j.1468-2370.2011.00324.x

